# How the Struggle for Public Health in the Jim Crow South Reflected and Reinforced Systemic Racial Health Inequality

**DOI:** 10.1017/s1742058x24000092

**Published:** 2024-10-31

**Authors:** Cheryl Elman, Kathryn M. Feltey, Barbara Wittman, Corey Stevens, Molly B. Hartsough

**Affiliations:** 1 Social Science Research Institute, Duke University and Department of Sociology, The University of Akron, Akron, OH, USA; 2 Department of Sociology, The University of Akron, Akron, OH, USA; 3 Lucy Cavendish College, University of Cambridge, Cambridge, UK; 4 Department of Sociology, Southern Illinois University, Edwardsville, IL, USA; 5 Department of Criminal Justice and Consumer Sciences, Youngstown State University, Youngstown, OH, USA

**Keywords:** Racial Health Disparities, Public Health, Community Health, Structural Racism, Jim Crow South, Rockefeller Sanitary Commission

## Abstract

The twenty-first century COVID-19 epidemic revealed a U.S. public health system that countenanced health inequities and a U.S. public that resisted disease containment policies. This crisis, however, was only the most recent chapter in a longer struggle in the United States to institutionalize public health. We focus on two early twentieth-century public health campaigns in the American South, the unhealthiest U.S. region at the time. Black southerners—denied basic health, political, economic, and social rights under a rising Jim Crow regime—self-organized health services networks, including through the Tuskegee Woman’s Club, the Negro Organization Society of Virginia, and the Moveable School (1890s–1915). Around the same time, a philanthropic project, the Rockefeller Sanitary Commission (RSC, 1909–1914), seeded state-level public health agencies in eleven southern states, thereby installing public health in a top-down manner. We use archival data sources to explore key similarities and differences in the public health concerns and coalition-building approaches of each campaign and southern resistance to their efforts. We find Black-led campaigns often blurred the color line to form coalitions that provided services to the underserved while tackling environmental health risks at the community level. In contrast, RSC affiliates in southern states, as directed by RSC administrators, provided health services as short-term public dispensaries. Services reached Black and White communities willing to participate but in a manner that did not overtly challenge Jim Crow-era practices. Southern resistance to public health expansion persisted under each approach. The legacies of these struggles remain; the political-economic and ideological forces that limited public health expansion while marginalizing Black community health efforts reverberate in public health inequities today.

## Introduction

The COVID-19 epidemic that reached U.S. shores in 2020 provoked (re)discovery of the importance of—and often inadequacy of—national, state, and local public health policies (Bonilla-Silva 2022; Editors NEJM 2020). Rapid disease spread revealed public health practice gaps and communication problems (Editors NEJM 2020). Health officials faced public resistance if not outright hostility while mobilizing communities to contain disease (Hotez 2021). Disproportionately high mortality rates in communities of color revealed long-standing disease pathways associated with structural racism: worker concentration in high-risk front-line jobs; neighborhood segregation and household crowding; unequal health system access and treatment (Bonilla-Silva 2022; Goodman and Schneider, 2023; Lundberg et al., 2023; Richardson et al., 2021; Tan et al., 2022). In response to racial/ethnic disparities in COVID-19 mortality rates, national public health officials expanded service coverage by partnering with local community-based groups in more inclusive ways. As national COVID-19 precautions lifted in 2023, however, there was not yet a consensus in the public health community or the public-at-large about what an all-inclusive “public health” might constitute and how it might be advanced, going forward.

As this article will show, the COVID-19 crisis is only the most recent U.S. chapter in a century-old struggle for public health. We examine two early twentieth century public health campaigns in the American South. One campaign was fueled by grassroots activism among southern Black-led neighborhood, university, religious, and professional groups between the 1890s to early 1900s. By 1915, they had forged organizational alliances that reached across and beyond the South, as a Sanitary Reform Movement. The second campaign, the Rockefeller Sanitary Commission for the Eradication of Hookworm Disease (hereafter RSC), was a five-year philanthropic project funded with a $1 million gift by John D. Rockefeller that recruited eleven southern states: Alabama, Arkansas, Georgia, Kentucky, Louisiana, Mississippi, North Carolina, South Carolina, Texas, Tennessee, and Virginia. The project commenced in October 1909 and it formally (and abruptly) ended in December 1914, leaving behind fledgling, state-level public health agencies (Ettling 1981; Farley 2004; Link 1992).

Our study uses primary historical data sources, including newspaper and journal articles, documents, images, letters, and recorded interviews from the period (Lipartito 2013), and proceeds as follows. The section following the Introduction describes the southern health environment at the twentieth-century’s turn and the key concerns that motivated each campaign. Concerns overlapped and differed. For example, Black-led groups and the RSC mobilized in response to the poor sanitary conditions in the American South. But Black public health activism was further rooted in a broader understanding of health as a social right (Smith 1995), while the RSC set out to improve states’ administrative public health capacity as much as their sanitation (Elman et al., 2014; Ettling 1981; Farley 2004).

The third section more closely examines political, economic, and ideological barriers that limited early public health expansion in the American South and each campaign’s coalition-building activities in light of these barriers. As with health concerns, campaign strategies and activities overlapped and differed. For example, both public health movements proceeded under a rising regime, termed Jim Crow (Wacquant 2002), whose laws and practices buttressed a structural racism of segregation and political disenfranchisement, and an ideological racism that cast Black persons as bio-physiologically sick and culturally inferior (Bobo and Smith, 1998; Bonilla-Silva 2006; Darity and Mullen, 2020). While both campaigns sponsored environmental clean-ups, public health clinics, and education about community health risks, Black-led groups marshalled participation across social and racial divides while RSC service activities, self-described by RSC administrators as “without race distinction,” abided by Jim Crow practices. The fourth section describes how public health practices “without race distinction” in this era could still leave Black and poor southern communities at the very margins of public health. The fifth and final section discusses legacies of these historical public health struggles.

The purpose of this study is not to detail each campaign as an historical example of public health reform; the excellent studies that we cite do this well. Rather, our first aim is to provide exceptional, fine-grained descriptions of each campaign in the context of the other and the operations of both in the context of the turn-of-the-twentieth century Jim Crow South. We do this to achieve our second aim: to bring past struggles in establishing public health across color lines forward, to shed historical light on the current struggle to inaugurate an all-inclusive system of public health.

## Starting Points: Public Health Concerns

### Concerns of Black Health Reformers

At the twentieth century’s turn, the South was the unhealthiest U.S. region (Breeden 1988). It harbored high-fatality infectious diseases found across the nation, including smallpox and tuberculosis, and those found mostly in the South, such as yellow fever, malaria, and hookworm (Elman et al., 2014; Kunitz 1988; Humphreys 2009). Non-southern states, however, were first to take public health steps to reduce mortality rates from disease (Duffy 1990). In 1900, ten U.S. Death Registration states actively tracked disease outbreaks and mortality^1^ but only about 4% of the U.S. Black population lived in these states. About 90% and 85% of the U.S. Black population in 1900 and 1920, respectively, lived in southern states (Hobbs and Stoops, 2002). The southern states, at best, minimized the need for public health; at worst, they resisted it (Beardsley 1987; Humphreys 2001; Smith 1995).

Hence, one concern of early twentieth-century Black health reformers was that public health reliance on national mortality statistics, primarily based on U.S. Death Registration data, masked the scale and suppressed the determinants of mortality in southern, primarily-rural Black communities. Current studies link early- to mid-twentieth-century Black-White differentials in infant, child, and adult mortality to Jim Crow-era racialized social structures and discriminatory practices (Elman et al., 2023; Ewbank 1987; Franklin and Wilson, 2020; Humphreys 2001; Karbeah and Hacker, 2023; Krieger et al., 2014; Logan and Parman, 2018; Zelner et al., 2017). But the high-profile studies of the time, based on Registration state data alone, pronounced that “color and race are possibly not the most important factors in the production of the relatively high mortality rates of the colored population of the United States” (Trask 1916, p. 257).^2^ Virginia physician Dr. David Byrd, a founding member of the Black Physicians’ National Medical Association and former consultant to the U.S. Surgeon-General, countered:

the Department at Washington, for the purposes of comparison with other nations, seeks a section of the country devoid of Negro population and declares it is necessary so to do (sic) because of our great maternal and infant mortality. We most respectfully urge the Department to study the death rate in the black belt and use its wealth and science to ameliorate conditions making possible this great rate

(Byrd 1917, p. 177).

Dr. Byrd here pointed to the “black belt” or the rich-soiled Plantation South—where most of the U.S. Black population lived and then died, uncounted—as the best place to study Trask’s (1916) “important factors” in high Black mortality rates.^3^ But Dr. Byrd and other Black community- and university-based health reformers were well-aware of the “important factors” and the “conditions” associated with mortality across the Plantation South (Beardsley 1987; Smith 1995). Reformers, as early as the 1890s, provided health outreach to plantation districts (described in the next section) and they attributed high Black mortality rates to malnutrition, the lack of sanitation and clean water in plantation-provided housing, and a lack of health care (Brown 1914; Beardsley 1987; Humphreys 2001; Smith 1995).

Black-led community and professional groups were additionally concerned about distributive inequality in publicly-funded sanitary benefits (Boris 1989; Judson 1999; Smith 1995). Distributive inequality partly reflected the southern lag in public health: there was little to distribute. Southern states in 1900 lacked the financial resources of the Registration states to expand governmental functions (Breeden 1976, 1988; Link 1992). But southern policymakers were also disinclined to expand governmental functions, especially for public health (Elman et al., 2014; Link 1992). Southern legislatures of the period limited, often constitutionally, state-level executive power (James 1988).^4^ As Black voters were also disenfranchised (Perman 2001), limitation of state-level power left considerable power and control in local White government hands (James 1988; Malczewski 2011; Werum 1999). Local White elected officials were arbiters of public resource distributions and they not only limited public expenditures but rarely allowed expended dollars to reach Black communities (Anderson 1988; Malczewski 2011). Dr. A. M. Brown, President of the Black physicians’ National Medical Association in 1914, linked poor Black population health to distributive inequalities in the built environment, developed with public funds. His Presidential Address encouraged greater Medical Association involvement with public health legislation:

It behooves the State association to interest itself in legislation regulating the building of houses, the erecting of tenements, the screening of windows and doors, the keeping of environment, improvements of the streets and alleys, the furnishing of water, the dispensing of sewerage and the maintenance of parks

(Brown 1914, p. 221).

Southern White physicians, including public health physicians, further contributed to distributive inequalities in public health resource flows. White physicians believed that they best understood southern health needs, including those of Black persons (Breeden 1976; Fett 2002). But most adhered to racist ideologies circulating at the time, including beliefs that inherently-unhealthy Black persons would not survive freedom, whether public health was available or not (Beardsley 1987; Breeden 1976; Patterson 2009; Smith 1995). White physicians might collectively support public health expansion but also might, as enfranchised citizens, vote to withhold tax dollars for sanitation projects benefitting Black communities. They might refuse to support higher education in Black communities, foreclosing health professions training for Black youth (Anderson 1988). They might further block Black American professional practice in publicly-funded health care facilities (Gamble 1995; Thomas 2011). Dr. Charles Roman, a Tennessee physician and first editor of the *Journal of the National Medical Association*, wrote:

Why should not the colored people have their proportionate share of public money for educational and sanitary purposes? Why should not colored nurses, internes and physicians be in control of the colored wards of public hospitals? Why should strictly colored communities not have colored sanitary inspectors?

(Roman 1917, p. 66).

We recall that Dr. Byrd argued that federal efforts to study and ameliorate conditions in the “black belt” of the Plantation South would prevent deaths of Black men, women, and children. He called for what southern White policymakers of the time would most detest: federal intervention. As a former consultant to the U.S. Surgeon-General, Dr. Byrd understood—as did other Black public health reformers—that influence from outside of the South might pressure southern officials to pass public health measures that they otherwise would reject (Smith 1995). Critically, this was the very same insight of RSC Administrative Board members: southern public health might be best advanced through pressure and incentives originating from outside of the South. In the next section, we turn to RSC concerns. Following this, we describe how, in the face of southern resistance to public health, RSC project affiliates and Black community health activists marshaled support for their respective public health campaigns.

### Concerns of RSC Philanthropists

All southern State Medical Societies between the 1870s to 1890s supported the establishment of Boards of Health. State legislatures provided the Boards with quarantine powers—but not necessarily the funding—to respond to periodic epidemics of yellow fever, smallpox, and other infectious diseases (Allen 1943; Ferrell 1932; Humphreys 2001; Porter 1999). Some State Boards managed epidemics better than others. For example, the Tennessee legislature in 1877 authorized but did not fund Board of Health activities; they added funding in 1879 but only for fighting epidemics.^5^ Neighboring Arkansas legislators directed their Governor to form a Board of Health in 1880 and also did not provide funding; they did not re-visit the issue until 1911, when legislators again rejected funding. Given this patchwork public health coverage, when an 1896 smallpox epidemic arose in the Arkansas portion of the Mississippi Delta, U.S. Public Health Service officers fought its spread from neighboring Tennessee. A.C. Smith, Assistant Surgeon Inspector wrote to the Surgeon General:

Sir: I have inspected part of Crittenden County, Ark…There are 24 cases of smallpox in 4 house-holds; 9 recovered, 5 died, 7 convalescent, 3 recent mild cases; no health officer; local physicians will not visit cases; 4 families require quarantine; can best be quarantined on plantations; county judge will cooperate and maintain quarantine… have to make Memphis headquarters

Smith (1970 [1896], p. 63).

In the meantime, residents of Tennessee and other states fled to Arkansas to escape their own epidemic outbreaks. Lizzie Barnett, a survivor of Tennessee plantation enslavement recalled a Yellow Fever epidemic:

While I lived in Memphis, da Yellow Fever broke out. You have never seen the like. Everything was under quarantine…Yes the times were aweful in Memphis enduring the plague. Women dead lying around with babies sucking their breasts. As soon as the frost came and the quarantine was lifted I came to Conway.^6^

State and local governments, lacking full-time health officers, primarily appointed part-time MDs, in active private or medical school practice, to respond to epidemics (Ferrell 1932). The MDs, however, might not conduct public health functions of reporting or even quarantine. United States Marine-Hospital Service Passed Assistant Surgeon C. P. Wertenbaker reported from Natchez, Mississippi:

There is a strong sentiment throughout all this section in favor of suppressing all information in regard to yellow fever on account of the injury to business and the possibility of a quarantine following such a report… a physician who would report the existence of a case of yellow fever is looked on and treated as an enemy to the place, with the consequent discomfort and loss of practice

(Wertenbaker 1900, p. 2902).

Yet, by 1900, the U.S. Death Registration states were finding better ways to control epidemics than quarantine, including by regulating and enforcing sanitary standards and mandating the reporting of diseases. Many non-southern states that lacked the financial means or administrative capacity to self-manage such improvements were accepting coordination, guidance, and/or financial assistance from federal agencies such as the U.S. Department of Agriculture, U.S. Public Health Service, and/or private philanthropic foundations (Beardsley 1987; Humphreys 2001; Thomas 2011). But no southern state before 1909 had established a state public health agency (at least one full-time officer) with ongoing surveillance and regulatory power (Allen 1943).

The South stood out to the RSC: it had high rates of hookworm, malaria, and other diseases and, considering public health expansion in U.S. Death Registration states, it was rapidly falling behind the nation in disease control through prevention. While the RSC was concerned about hookworm, its broader objective, as stipulated in RSC bylaws,^7^ targeted southern states as “units of organization and of work” for the purpose of creating permanent state-level administrative offices to regulate sanitation and monitor public health (Rockefeller Sanitary Commission 1911-1915, First Annual Report, p. 3). The RSC Administrative Board believed, as did U.S. Public Health officers, that disease prevention and containment required state-level regulation with full-time personnel for enforcement. Assistant U.S. Surgeon General Trask, visiting the first group of newly-installed public health officers in Arkansas, explained:

The people of Arkansas have, through their legislature and State board of health, made it a misdemeanor for a physician to fail to report to you every recognized case of certain designated diseases among his patients, and have fixed as a penalty for such failure a fine of not to exceed $100, or imprisonment for not to exceed one month, or both fine and imprisonment

(Trask 1913, p. 2528).

Trask also explained to the new health officers that they could move beyond containment to prevention of diseases “not limited to the communicable diseases…[and] due to improper living or working conditions” (Trask 1913, p. 2527).

Certainly, hookworm, the helminth *Necator Americanus*, was of RSC concern. The worm became a national celebrity in 1902 after Dr. Charles Stiles, a federal public health zoologist, labeled it the “Germ of Laziness,” responsible for the South’s lack of economic development, relative illiteracy, and impoverishment (Elman et al., 2014; Ettling 1981).^8^ Hookworm could not compete with Dr. Roman’s “lethal diseases,” but an attack on the notorious worm could quickly gain the philanthropists sympathetic national attention and allow them to spotlight the dangerous sanitary conditions in the rural sections of the South. As many lethal diseases thrived under the same poor sanitary conditions as hookworm, improved sanitation, even as self-contained outdoor privies, would reduce population risk of lethal disease (Ferrell 1913; Link 1988; Stiles 1915). While hookworm was less-often fatal, it contributed to mortality in concert with other diseases and health deficits such as malnutrition. Even the best-trained medical doctors and public health specialists of the time might not be able to distinguish between southern diseases in the field—a prerequisite for reporting, containment, and reducing spread. During his visit to Natchez, Mississippi, Passed Assistant Surgeon Wertenbaker reported:

I met Dr. H. A. Gant, president Mississippi State board of health, who had come over from Jackson, Miss…[to investigate] a fever, diagnosed as dengue by some physicians, and pernicious malaria by others… About November 1 Dr. Gant visited Natchez to ascertain if the disease was yellow fever, but…[he] was unable to make a decided diagnosis

(Wertenbaker 1900, p. 2901).

RSC affiliated MDs, once in the field, further reported that:

a very large per centage [sic] of persons suffering from malaria are suffering also from hookworm disease…it is practically useless to try to cure chronic malaria until the patient is cured of hookworm disease…[also] people who are suffering from hookworm disease are more subject to typhoid, tuberculosis, pellagra, malaria, and pneumonia.^9^

RSC administrators believed that an attack on hookworm might lead to medical understanding of hookworm and other then-mysterious tropical diseases of the United States and Global South, including as co-morbid and syndemic conditions (Humphreys 2009; Tsai et al., 2017).

RSC administrators were additionally concerned about hookworm’s effect on labor productivity in the American South and the Global South. Indeed, as the RSC focused on hookworm in the American South, another, concurrent Rockefeller-funded project was assessing hookworm prevalence in plantation-holding Global South countries. The Rockefeller-funded International Health Commission (IHC), in the planning stages prior to 1913, was launched in 1913 with overlapping RSC and IHC Administrative Board memberships (Farley 2004).^10^ The American South was one small piece in a larger, international puzzle.

Project scientific rigor was another RSC concern, especially after 1911, including how to best conduct: (1) mass hookworm screening and treatment in community settings and (2) survey community privy (outhouse) quality in dwellings, schools, and other public places. RSC project affiliates, initially instructed to visually surveil local populations for evidence of disease, found this visual diagnostic method inadequate. In 1911, the RSC initiated school-based hookworm prevalence surveys that used microscopic techniques to estimate parasite burden in rural children. The surveys found hookworm prevalence to be around 40% in southern rural children.

Wickliffe Rose was appointed Executive Secretary of the RSC Administrative Board in January 1910 and he brought his own concerns and experience about education and public messaging to the project (Farley 2004). A Tennessee educator, Rose additionally served as Executive Secretary of another Rockefeller-supported Board, the General Education Board (GEB, formed in 1902),^11^ and, after 1913, as IHC Executive Director. As RSC head, Rose guided project activities in non-medically-oriented directions (Ettling 1981; Farley 2004), describing the project as:

an educational campaign reaching from 20,000,000 to 25,000,000 [southern] people in the interest of better sanitation. While the campaign is directed specifically against hookworm disease the same sanitary measures that are being urged will go a long way toward eradicating amoebic dysentery, Cochin China diarrhoea, typhoid fever, and other enteric diseases.^12^

In early 1910, Executive Director Rose began appointing and funding full-time State Directors and staff in the eleven southern states. The RSC required participating states to hire RSC-approved State Sanitary Directors and staff to State Health Boards. Cooperating states thus took first steps in building state-level public health administration.

## Expanding Public Health: Two Approaches

### A Reach into the South: RSC Philanthropic Efforts

RSC administrators planned to quickly appoint State Directors and hire field staff in each of the eleven southern states. They expected State Directors and their affiliates (hereafter, RSC affiliates) to fan out across their respective states to estimate hookworm prevalence. RSC administrators further understood that hookworm “eradication”—as the project’s title promised—could only succeed with southern White elite cooperation as southern plantation landlords (hereafter planters) and industrial employers owned most of the housing stock used by Black and White tenant farm and mill-town employees (Beardsley 1987; Humphreys 2001; Smith 1995; Stiles 1912). Most of these southern dwellings lacked indoor or even sanitary outdoor privies, as did most if not all schools, commercial buildings, churches, and public institutions (Beardsley 1987; Stiles 1939). To ensure project cooperation, RSC administrators charged RSC affiliates with finding collaborators among elected officials, physicians, school organizations, community groups, and the local press (Ettling 1981; Farley 2004; Stiles 1939).

But historical context is important. By 1910, southern White planters and White urban and business elites had consolidated political power in single-party Democratic rule (Ayers 1998; James 1988; Perman 2001).^13^ Elected Democratic state legislators and county officials primarily won elections by promising lower taxes, at least for planters and other property-owning farmers, and fewer regulations for local businesses (Ayers 1998). Additionally, many southern White physicians harbored sentiments of “states-rights medicine” that had emerged in the run-up to the Civil War; they especially resented northern medical community claims of superior medical education, training, and practice (Breeden 1976, p. 368).^14^ More generally, under prevailing southern White localism and traditionalism, especially in rural areas and small-towns, non-southern “outsiders” were not trusted (Link 1992; Schulz 2005). It was in this context that RSC affiliates set out to build support from southern White elected and professional elites who, at least initially, viewed RSC-sponsored public health efforts as meddlesome—if not invasive and insulting—and expensive (tax-based) (Link 1992; Stiles 1939).

Not surprisingly, the RSC’s initial reception at the state level was markedly uneven. In March 1910, Executive Director Rose appointed Dr. John Ferrell as RSC Health Director in North Carolina, a health-progressive state. Dr. Ferrell conducted hookworm activities in nearly sixty of 100 North Carolina counties before 1912. Kentucky’s Dr. Arthur McCormack, appointed in 1911, reported that his own appointed affiliate, Dr. Richmond, spent his first six months reaching out to “…Medical Societies, newspapers and teachers, as well as the public”^15^ to assess interest in RSC activities. Texas did not accept RSC support until 1912 because previous Governor Campbell (1907–1911) believed “Texas can better endure the hookworm than lay itself debtor to Rockefeller.”^16^ State Attorneys General in South Carolina and Mississippi barred counties from supporting RSC activities but they eventually backed down.^17^ Arkansas, the most resistant of the eleven states, blocked state-supported RSC activity until 1913, a year before the project ended. Arkansas RSC-funded Dr. Morgan Smith notified Executive Secretary Rose in 1911:

A most unfortunate condition exists concerning our Public Health Bill…inasmuch as there had been such violent opposition manifested by certain influences, I traced the Bill from the House after its final passage to the Secretary of the Senate…all trace of it being lost… [then] with but a few hours left…I scoured a printed copy of the Bill…[it] was presented to the Governor who signed it without question…we all went out in the air and took a long breath—the end of the hardest fought battle ever waged in this State.^18^

But soon afterwards, Dr. Smith notified Executive Secretary Rose that:

The Attorney General has advised… [the bill] differed in a minor detail from the original, and therefore…“the legislature did not pass the bill”…the state has no public health organization…[and it] will be two years yet before the disability can be relieved by legislation.^19^

RSC affiliates’ activities were further complicated in early 1911 when the RSC Administrative Board re-focused the project. Executive Director Rose learned that a short-term local dispensary, opened in Marion County, Mississippi on December 17, 1910, drew large and enthusiastic crowds.^20^ Rose soon contacted other State Directors and instructed them to set up short-term public dispensaries in all counties in their respective states (Farley 2004). The dispensaries, generally six-week “one-stop shops,” provided clinical microscopic screening of hookworm ova in stool samples, facilitated treatment for those with positive tests, and educated attendees using lectures and microscopic hookworm displays. The dispensaries could also shift public attitudes about a “new scientific conception of disease…that germ theory could bring,” at least in the short run (Kunitz 1988, p. 143; also, Duffy 2011). As an RSC affiliate reported:

Every progressive citizen, when he looks through the microscope and sees the living larvae of the hookworm, as can be done, and is being done every day by hundreds of people, is convinced that…47% of our school children are continuously spreading the disease by non-use of privies.^21^

The dispensaries, however, complicated the work of RSC affiliates. Rockefeller-funded philanthropies never equated their philanthropy with charity (Anderson and Moss, 1999; Ettling 1981; Farley 2004) and RSC administrators required “all local [dispensary] expenses [to be] born by county funds” (Rockefeller Sanitary Commission 1911–1915: Second Annual Report, p. 18). While Rose commended Mississippi State Director Leathers on a successful first dispensary, he also informed Leathers that RSC project funds would not cover dispensary costs in Mississippi or any other RSC state. Rather, Rose noted that “every county in the South could well afford to put up the money necessary for the eradication of this disease until the job is thoroughly done.”^22^ Thus, RSC affiliates—busy cultivating general state-wide project support—needed to also court county officials to approve and pay for dispensary work.

RSC affiliates quickly became embroiled in a southern patchwork of local jurisdictions where White County executives, judges, and boards wielded veto-power over public health. Shown microscopic and statistical evidence of local hookworm infection, county officials might refuse to: acknowledge a hookworm problem, contribute funding toward dispensaries, pay in advance and/or honor local-issued receipts for invited services rendered. In mid-1913, after the Arkansas legislature passed its Board of Health Bill, State Director Dr. Garrison (RSC-Arkansas, Dr. Smith’s replacement) complained to North Carolina’s State Director Ferrell:

[In Arkansas] most of the County Judges are not disposed to allow appropriation until the work is done and an itemized account submitted…I have advanced…cash and stand to lose…I shall not attempt work in any more counties until I am assured by the County Judges and Quorum Court that I will be repaid.^23^

Dr. Ferrell, working in a more health-progressive state, replied:

Different methods are followed in different counties. But the field directors are seldom called upon to advance their personal funds…As a State Director I have never had to trouble myself about securing money from the counties.^24^

Yet even RSC affiliates in progressive North Carolina faced resistance: obtaining an appropriation from Alamance County was “a hopeless undertaking…shunned by other directors” and only occurred when Dr. Washburn (NC) targeted health-progressive county officials, one-by-one:

[M]y first interview was with the county attorney…from him I learned the characteristics of the members of the board of county commissioners. There were five commissioners: two were progressive, one decidedly unprogressive, and two neutral

(Washburn 1914, p. 2).

Local White officials, elected on a promise of low taxes, might themselves support sanitary work but their enfranchised White constituents—wielding the power of the vote—might retaliate. RSC-appointed State Director Dr. Olin West of Tennessee, worried about voter backlash, requested that the RSC keep sanitary improvements secret:

You will see in our quarterly report that Clay, Madison, Scott, and Davidson counties are committed, through their Boards of Education, to the building of sanitary closets for their schools. Some of the Boards do not want advertisement of this action on their part. They fear that publicity will raise a “howl” upon the part of taxpayers.^25^

Some county officials rejected public health interventions for hookworm specifically. Albemarle, NC County Commissioners rescinded a dispensary appropriation, explaining:

It would be the ruin of our party if news got out that we are spending people’s money for such a thing as hookworm treatment…people here are of a different breed from those in other places – they won’t fix these specimens in your little tin boxes.^26^

Some White physicians ideologically resisted, hence fiercely contested, *public health* as opposed to private medical practice (Beardsley 1987; Kunitz 1988). Tennessee Director Dr. Olin West fumed about a published article that insinuated “high-cost” public health work was unnecessary and wasteful:

Statements…to the effect that “twenty-five cents worth of medicine” can be expected to cure hookworm disease and bring about great indirect benefits are to be deplored…men whose influence and cooperation we need and seek tell me that they have read articles like that…[they] will no longer listen to our appeals.^27^

Yet, public interest in RSC dispensaries quickly spread across counties, in snowball fashion, including Black communities. Executive Director Rose wrote to Alabama State Director Dinsmore:

The work being done thus far in all the states has been, as I understand it, without any race distinction…I think I have never visited a dispensary without seeing both races present…The sanitary surveys as you know are made without race distinction. These surveys are reaching both races and I am inclined to think is reaching them rather effectively. The practicing physicians of both races are being enlisted.^28^

Rose uses language suggesting considerable RSC racial inclusion, for the period, in clinical and educational activities. We return to this after examining Black-led groups’ coalition-building approaches amidst southern resistance.

### Grassroots Movements across the South: Black-Led Campaigns

The RSC fielded a top-down, tightly administered campaign (Cairncross et al., 1997) using RSC affiliates to conduct surveys and peel away enough White leadership support to authorize and fund county-level short-term dispensaries. In contrast, Black-led groups formed broad-based coalitions that slowly expanded community coverage of health activities from local levels in the 1890s to early 1900s, to statewide and national campaigns in the 1910s to the 1950s. Less hostile to “outsider” social connections, they partnered with White local groups and/or elicited philanthropic and/or governmental support for inclusive social and health projects (Brown 1937; Judson 1999; Smith 1995).

Historical context is again important. Black-led mobilizations for public health arose at a historical inflection point: at nadirs of Black population health and race relations under a rising Jim Crow South (Beardsley 1987; Smith 1995). Black communities that were losing or had lost political, economic, and social rights under Jim Crow rule (Darity and Mullen, 2020) viewed their right to a healthy environment as part of the political, civil, and social rights struggle (Gordon 1991; Judson 1999; Smith 1995). For Black activists, grounded in belief of racial solidarity, “the primary launching site of every struggle was the community” (Hine 2007, p. 2). At the same time, much of their work involved institution-building within their communities, including the “schools, old people’s homes, medical services, community centers…[that] the white state would not” (Gordon 1991, pp. 560-561; also, Anderson 1988). Black communities across the South raised the additional funds for this institution-building through self-taxing, after paying required taxes for segregated public institutions (Anderson 1988).

Black university and urban groups were among the earliest to organize and many provided outreach to the rural Plantation South. In Alabama, the Tuskegee Woman’s Club initiated health and social welfare projects in the late 1890s (Smith 1995). An annual Tuskegee Negro Conference had addressed issues of poverty and self-help in 1892 (Campbell 1969 [1936]) but the Woman’s Club went beyond discussion to founding a settlement house. Volunteers soon traveled off-campus, visiting nearby plantations where Black convict-release sharecroppers lived in planter-provided housing with minimal—if any—sanitation, clean water, or access to high-quality food (Dirks and Duran, 2001; Smith 1995). Visiting Club members provided clean-up demonstrations and advice on “how to prepare and serve their food…[and also about] tuberculosis and typhoid fever.”^29^ Howard University’s Alpha Kappa Alpha Sorority, founded by Black students in 1908, later organized and funded medical teams providing summer medical care for children and others in the rural Mississippi Delta (Gamble 2016; Smith 1995).

In Georgia, the Atlanta Neighborhood Union, a Black women’s organization founded by social reformer Lugenia Burns Hope in 1908, began as a charitable institution committed to the welfare of Black women and children. After opening a settlement house, the Union addressed sanitation, nutrition, and disease in Atlanta’s most underserved Black neighborhoods (Judson 1999; Smith 1995). In 1912, the Union surveyed Black school health problems and, in 1919, it mobilized a contingent of over one thousand volunteers to survey plumbing and sanitation (privies) in thousands of neighborhood dwellings (Gordon 1991; Judson 1999; Smith 1995). The Union’s anti-tuberculosis campaign pre-dated the Atlanta Anti-Tuberculosis Association (ATA) formed by White women with whom the Union then partnered in 1914 (Judson 1999; Thomas 2011).^30^ In 1915, the Union opened a health clinic, then continued outreach activities by establishing mobile health clinics in Black neighborhoods. By 1930, thousands of Atlanta’s Black citizens relied on Union clinics for their basic health care needs (Judson 1999).

In this Progressive Era, more generally, White as well as Black women’s groups sought social and public health reform. But, emblematic of the structural racism of the time, White women’s clubs, members of the General Federation of Women’s Clubs founded in 1890, prohibited Black women from joining (Boris 1989; Neverdon-Morton 1978). In response, Black women formed their own National Association of Colored Women in 1896, although they “organized separately but not as separatists” (Boris 1989, p. 32). It is also emblematic that, despite shared concerns about health and social welfare, the clubs differed in their proposed approaches to public health reform (Gordon 1991). White women’s clubs sought to reform individual and household hygiene, or the “private side of public health,” and called for means-tested services (Tomes 1998, p. 6). Black women’s clubs—more concerned about distributional equity and universal environmental solutions, such as community-wide access to clean water and sewage disposal—pressured local officials to redirect public spending to address systemic causes of poor health (Boris 1989; Judson 1999). To achieve their goals, they strategically used White fears—that diseased Black populations might threaten the health of White persons—in their work with White reformers and politicians (Judson 1999).^31^

Black men’s public health activism also included rural outreach and followed expansionary paths of growth (Beito and Beito, 2006). The Men’s Sunday Club of Savannah, established in 1905 as a social network of Black and White church members, medical professionals, business groups, and interested individuals, promoted sanitary improvement and provided health-related services. In Virginia, the Negro Organization Society, a community-building self-improvement alliance under a banner of “better farms, better homes, better education, and better health,” held a Clean-Up Day sanitation drive in 1912 that expanded to a week in 1913, drawing 130,000 Black and White Virginia participants (Beardsley 1987, pp. 102-103). The Negro Organization Society, itself, grew to include 250 Black religious and secular organizations, representing almost half of Virginia’s Black population (Brown 1937; Smith 1995). The Tuskegee Institute adopted this initiative and in 1915 launched National Health Improvement Week (Quinn and Thomas, 2001).

Some Black-led organizations coordinated with philanthropic foundations and/or accepted government support. For example, George Washington Carver and Tuskegee Institute students, with Rockefeller-funded General Education Board (GEB) and federal Department of Agriculture support, created a multi-decade rural outreach program, initially using a “Jesup Agricultural Wagon” as a Movable School of Agriculture (1906). The early Movable School wagon visited rural areas of Macon, Georgia to demonstrate farm, nutrition, and health techniques (Mayberry 1991; Smith 1995). In 1914, with Alabama Polytechnic Institute support (now Auburn University), the Movable School traveled to neighboring counties and implemented these visits as Demonstration Days.

Prior to a Demonstration Day visit, Movable School project workers assessed the availability of a teacher, preacher, or officer of a local church in the community as a point of contact. Once a contact was made, project workers located an area farmer or obtained permission from a local planter to use a tenant farm, to:

[be] a “classroom” during the period of the school…his home will be improved by members of the community through having …work done, such as screening the house, covering the well, painting the outbuildings, constructing a sanitary toilet, setting out an orchard

(Campbell 1969 [1936], p. 118).

Each festive Demonstration Day might attract hundreds of farmers, plantation tenants, and family members. The original mule-drawn Jesup Wagon visited over 2000 Black farm households in its first few months of use. Seventeen years later, in 1923, 30,000 Black farmers purchased the truck for Moveable School visits that included health instruction by nurses (Campbell 1969 [1936]; Smith 1995).

Over time, Black-led campaigns networked regionally and nationally. The Movable School continued to 1944, extending Demonstration Days to neighboring states. Negro Health Week became an annual program with national scope and continued to 1951. Howard University’s Alpha Kappa Alpha Sorority provided Mississippi Delta medical care to the 1940s (Smith 1995). Professional groups of “colored nurses, internes and physicians” (Roman 1917, p. 33) also mobilized across the South. By 1920, a Black physicians’ hospital movement was fighting for the right to practice medicine in public hospitals (Gamble 1995) while primarily-Black southern midwives mobilized for recognition, education, and access to resources (Sano 2019; Smith 1995).

Like RSC affiliates, Southern Black public health reformers faced generalized southern resistance to public health expansion and regulation. They, like RSC affiliates, might respond to White elites’ diminution of surrounding toxic environments and high death rates with economic logics of persuasion. In his travels across the South, RSC Administrator Stiles proclaimed that removing “soil pollution is not only in the interest of the public health, but also in the interest of the further economic development of the South, especially on the farms and in the textile industries” (Stiles 1909, p. 1447). RSC State Director Dr. Garrison of Arkansas warned that malaria, alone, cost near-insolvent Arkansas from $12 million to $16 million yearly in economic losses.^32^ Dr. A. M. Brown warned, in the *Journal of the National Medical Association*:

sickness and death cost the Negroes of the South alone one hundred millions of dollars annually…450,000 Negroes are seriously ill all the time and…one hundred thousand of these lives can be saved… fifty millions of these hundred millions of dollars…could be applied on Negro schools

(Brown 1914, p. 220).

Black health reformers additionally faced public health resistance rooted in the Jim Crow-era economic laws that advantaged White planters and disadvantaged Black tenants in agricultural tenancy arrangements (Fields 2001; Woodman 1995). Much of the Black population over the first decades of the twentieth century lived under “peonage in the rural South… buttressed by a system of statutes and a body of legislation positively astonishing in its reactionary and mediaeval aspect” (Du Bois 1912, p. 82). Thomas Monroe Campbell (1883–1956), an early pioneer in the fields of agricultural education and health extension work at the Movable School, witnessed:

Approximately 50% of the rural Negro boys and girls are children of parents who each year mortgage all of their personal property and the labor of their families for funds or provisions with which to make the current crop. This practice binds the whole family

(Campbell 1969 [1936], p. 148).

Critically, Black health reformers, physicians, and other practitioners such as midwives who provided outreach found that the families that they served most often *could not escape* the impoverishment and abysmal living conditions that threatened their health. These reformers were aware of the great “death rate in the black belt” (Byrd 1917, p.177) and its source: disproportionately greater exposure of impoverished Black tenant farm households—legally-bound, if not entrapped, in economic arrangements—to harsh living conditions (Smith 1995). Dr. S. B. Hickman, a Black physician in Tennessee, confronted a White planter:

I was asked not long ago by a large planter…why pellagra and malaria were playing such frightful havoc there. I thank God for the opportunity of looking him squarely and unflinchingly in the face…saying: “…There is not a single screen to be found on your plantation except those on your own house. There is not a well on your entire farm yielding decent drinking water. You sell them third-class foodstuffs at a firstclass price…You in your greed for money, have placed a value on cotton exceeding that of the human life”

(Hickman 1916, p. 143).

This planter eventually made improvements and Movable School organizers found White planters amenable to allowing tenants a Demonstration Day. But many more planters, aware of the high disease and death rates in their labor pools, made little effort to improve work and living conditions. Rather, they viewed these high rates among their workers as an economic cost of business. Arkansas State Director Dr. Garrison reported:

A manager of a large plantation in Arkansas estimated that because of malaria, he had twice as many tenants on the plantation as would have been necessary to run it if they had no malaria.^33^

Black health reformers additionally found southern resistance to public health—for Black communities—tied to racialized practices of social control (Steedman 2008). White planters, as medical gatekeepers, could refuse medical loans or permission to see White physicians if Black workers did not behave in a deferent manner or maintain employer goodwill (Schulz 2005). White employers might even ban Black workers’ access to Black community health workers. After Alpha Kappa Alpha volunteers set up a health clinic in Holmes County, Mississippi in the 1930s, excellent clinic attendance quickly dropped off because “white plantation owners, wary of ‘outside agitators’ refused to allow ‘their’ sharecroppers to leave the plantations and attend the clinics… (Smith 1995, p. 155).

Labeling Black health reformers—even if they lived locally—as “outside agitators” (Gamble 2016) was an additional racialized form of southern public health resistance, as was southern White diminution of a need for publicly-funded sanitation in Black neighborhoods. Black health reformers proposing this to officials might be informed that Black communities were inherently “sicker” and would not benefit from improvements or were spreaders rather than recipients of disease risk (Beardsley 1987; Patterson 2009; Smith 1995). Indeed, the most health-progressive White southerners then-gravitating toward germ theory more sought public health to protect themselves from disease across the color line, than to improve Black community or universal health (Anderson 2002; Beardsley 1987; Patterson 2009).

## “Without Race Distinction”

We recall, from Executive Director Rose, the absence of “race distinction” in RSC operations. Rose’s choice of words is interesting in the historical context of the Jim Crow South, as race was then a central organizing principle across political, economic, and social domains. Operating “without race distinction” would be an important harbinger of a future public health if it involved racial inclusivity in educational and clinical activities. To be clear, “without race distinction” for Rose meant:

[F]ield men have visited the schools of both races; have lectured to audiences of both races; literature is sent to physicians, teachers, and people of both races; the state laboratory is giving free examination of all specimens sent it without regard to race; the dispensaries examine and treat all people that come regardless of race.^34^

It is not possible to directly evaluate specific activities “without race distinction” because RSC administrators did not systematically collect data on educational participation, dispensary attendance, or clinical testing by race, even for private administrative use. However, RSC administrators—aware that project success required community support—quantitatively measured project success in terms of RSC affiliates’ general educational as well as clinical activities in schools and dispensaries (Link 1992). In order to evaluate this, the administrators required affiliates to tally and report events, including photographs. Administrators then published each state’s yearly activities in Annual Reports disseminated to national groups, southern elected officials, academics, and physicians as a public relations tool. These reports, along with prior studies’ findings and archival holdings, provide indirect indicators of racial inclusion across activities.

For example, published Annual Reports depict project activities that included Black and White attendees, but in a manner that would not challenge Jim Crow-era customs, politics, and power relations. Notwithstanding that Rose “never visited a dispensary without seeing both races present,” published photographs of dispensary, school, and other activities in the five Annual Reports allude to segregated groups and activities. Whether published photographs reflected segregated activities (e.g., all schools publicized would be segregated), selectivity in RSC affiliates’ photographic submissions, or in administrators’ publication choices, most RSC activities memorialized an expanding public health that did not challenge segregation practices. Also, RSC clinical and educational activities provided “without race distinction” were provided without Black physicians as RSC affiliates. Black physician candidates were available for RSC affiliate appointments but RSC administrators did not hire them in any participating state (Ettling 1981; Marshall 2015). The administrators were silent about this in archival material but Black physicians of the time were not. Dr. John Kinney of the Tuskegee Institute asked RSC State Director Dinsdale of Alabama to hire a Black physician as an RSC affiliate:

In a previous letter I asked if you thought it feasible or practical to put a colored physician in the field to go among the colored people… in a way hardly practical for the white physicians

(Jones et al., 1912, p. 310).

The issue of RSC employment involved distributional inequity, as a lack of “colored sanitary inspectors in strictly colored communities” was harmful to Black community health (Roman 1917, p. 66). We understand this, today, as a lack of health care provider diversity. Dr. Kenny’s request indicated that White physicians then-filling RSC affiliate roles could not or would not fill public health educational and practice gaps—very important RSC project objectives according to Rose—in Black communities. Moreover, in the longer run, the RSC administrative decision to not employ Black affiliates foreclosed the possibility that state-level Black public health administrators, going forward, would fill southern state-sanctioned public health roles.

RSC activities “without race distinction” also shed light on Dr. Boyd’s (1917) concerns about public health information-gathering: inequity in data collection, in this or any period, conceals health disparities and their underlying determinants. While public health studies of the time did not always differentiate demographic categories (Marks 2003), RSC abstention from at least cursory examination of race differences in hookworm prevalence is notable, given the philanthropy’s then-concurrent international project targeting hookworm in plantation-holding countries of the Global South. RSC administrators may have believed race differentials in the American South were minimal, or unimportant, or that their overt study might jeopardize White elite willingness to fund RSC work, but Black health practitioners, aware of RSC survey work, did want to know hookworm prevalence rates in their communities.^35^ President F. S. Hargraves, MD of the North Carolina Medical, Pharmaceutical, and Dental Association, at a 1911 North Carolina meeting, called on the National Medical Association to conduct their own campaign:

[T]here should be formed local associations….[with] three commissioners – one on tuberculosis, pellagra and hookworm – to report at the next annual meeting…We should not be discouraged or despair because we are poor or members of a young race or because we have not a Rockefeller foundation to aid in our investigation and research

(Medical Society Notes 1911, pp. 265-266).

The National Medical Association quickly formed a broad-based Commission, self-described in its 1912 Report as “keenly interested in the racial elements in the etiology of hookworm disease” and “the human outlook in regarding the problem” (Jones et al., 1912, p. 314). The Commission’s 1912 Report both commended and expressed disappointment in the RSC:

While it has not been possible, on the whole, [for the RSC] to secure separate statistics bearing on the examination for, and treatment of, hookworm disease among the white and colored races…[the RSC provided] clinical and microscopic… [and] free treatment… [that] members of our race have shared as freely as any of the white race… For this we rejoice and take courage, though feeling some slight disappointment that the racial standpoint was disregarded in the investigation

(Jones et al., 1912, p. 308).

The 1912 Report concluded that “the hookworm problem is a human and not a racial problem…[and] part of the larger problem of sanitation and preventive medicine” but it also indicated how programmatic public health services offered without race distinction might disappoint when they disregarded “the racial standpoint” of the self-interested communities served (Jones et al., 1912, p. 315).

It is also important that service provision for persons who show up at the door, even “without regard” to race, does not preclude inaccessibility and inequity. The RSC conditioned public dispensary work on county appropriations; studies report uneven, weak county dispensary support (Elman et al., 2014; Link 1992; Stiles 1939). For example, compared to 99% of North Carolina and 93% of Mississippi counties, only 53% (forty-three of seventy-five) of late-starting Arkansas counties appropriated dispensary funds by the project’s 1914 formal end. In five of eleven RSC states even smaller proportions of counties provided funding (25-26%) than late-starting Arkansas (Rockefeller Sanitary Commission 1911–1915, Fifth Annual Report, p. 34).^36^ Under RSC project design, county participation was selective: educational and clinical services were more limited in counties more resistant to paying (least health-progressive) or least able to pay (poorer) (Elman et al., 2014). Moreover, counties least likely to participate were least exposed to a dispensary “treatment effect” that might shift public attitudes and increase local administrative capacity for future public health expansion (Link 1992). Consequently, the RSC cast a narrower, more selective net of public health activities across the South than otherwise possible.

In sum, the RSC might operate “without race distinction” and, at the same time, conform to prevailing Jim Crow institutional, social, and ideological practices. They might provide public health without regard to race, even without intent to discriminate, yet leave room for public health where racial inequities could freely operate (Bonilla-Silva 1999). Meanwhile, Black-led groups were mobilizing local community networks to provide health services across state lines, including outreach to the most geographically distant and the most underserved. In this historical moment, the RSC not only looked past potential alliance with Black health reformers—as co-equals—but overlooked their effective grass-roots coalition-building techniques which could have complemented the RSC’s short-term, transactional, top-down approach (Cairncross et al., 1997).

## Ending Points: Two Visions of Public Health

Black-led groups and the RSC, operating in a geopolitical field where Jim Crow-era laws supported, indeed required, systemic racial discrimination and social exclusion, took different approaches to public health. Black health reformers channeled social reform activism that included public health, primarily working through the “essential institutions: families, churches, clubs, hospitals, and health clinics” (Gordon 1991; Hine 2007, p. 16) that they, themselves, had built in the decades following the demise of Enslavement (Anderson 1988; Judson 1999; Smith 1995). In contrast, the RSC inserted RSC-appointed State Directors and staff into newly-formed regulatory state health agencies with instructions to set up short-term, county-funded dispensaries to screen, treat, and educate those persons able to attend. Critically, southern resistance to public health expansion *persisted under each approach* at a time when breaking the impasse of southern resistance to public health was everything. As both campaigns understood, continued resistance to environmental improvement and disease prevention guaranteed hookworm endemicity and future epidemic waves of high-fatality diseases in the American South.

The southern resistance impasse partly persisted because Jim Crow-era laws and practices contributed to it and even strengthened it. Disenfranchisement blocked Black voter political counter-pressures for local sanitary improvements. Segregated schools blocked pipelines of community-focused “colored nurses, internes and physicians” (Roman 1917, p. 66) while segregated health care settings blocked their potential scope of professional practice, from service provision to professionally-powered collective action. Racialized economic laws initially designed to control Black farm labor in the post-Enslavement era (Du Bois 1912; Fields 2001; Woodman 1995) set the stage for broader twentieth-century southern business elite control of domestic and workplace farm, mill-town, and mining-town health environments for most Black and White workers (Beardsley 1987). Racist ideologies about biological differences allowed business elites to minimize, if not ignore, disease and death among Black and poor White workers and families (Patterson 2009; Wray 2006).

Breaking the impasse was difficult for Black reformers; prevailing racialized forms of political-economic exclusion and ideologies outweighed their ability to compel southern White elites to fund universal sanitation and other public health functions (Hammonds and Reverby, 2019; Smith 1995). For the RSC, project efforts failed to convince the most resistant local White leadership, who not only blocked (refused to fund) the short-term demonstration projects, but longer-term public health expansion in the South (Link 1992; Stiles 1939). Recalcitrant Arkansas state legislators who in 1911 fought “the hardest… battle ever waged” to defeat public health, lost the battle in 1913, but then won the war: Arkansas, along with Alabama, Georgia, and Texas, did not enter births or deaths into national Vital Statistics pools until after 1926. The southern lag in sanitary improvement and the racial gap in this improvement then persisted to and beyond the mid-twentieth century: southern Black Americans in 1940 were significantly less likely than non-southern Black Americans to have indoor latrines (DallaValle and Britten, 1942). Hookworm, the disease that set the RSC on course to install southern public health a century ago, persists today in majority-Black Lowndes County Alabama and other southern pockets because of lack of access to municipal sewage systems (Flowers 2022; McKenna et al., 2017).

Even shared concerns about poor environmental conditions did not unite the campaigns, to perhaps break the impasse. Among structural barriers precluding collaborative work, in addition to Jim Crow-era laws and practices, were different visions or approaches to public health. The early RSC project innovated by combining a biomedical approach—focusing on a single disease (e.g., hookworm) for elimination by medical diagnosis and treatment (Duffy 2011)—and a health promotion approach, using public education and messaging to motivate prevention or treatment (Kunitz 1988). But this vision proselytized an elitist and technocratic administrative structure, routed through state-local governments, far-removed from community and social inclusiveness. Rose wrote:

It is probable that in the course of time some special work will have to be developed among the negroes. I thoroughly appreciate what Dr. Kenney says, that there are some things which a negro can do for his own people more effectively than it could be done by the white person. Just what form this will take I cannot foresee.^37^

Taking this approach, the RSC did not directly challenge southern practices of Jim Crow racism (Bobo and Smith, 1997; Bonilla-Silva 2006). Not being a charity, they needed financial support from local officials, whose own electability was contingent on limiting public expenditures, especially for Black communities (Ayers 1998). Even the most sympathetic local officials that authorized dispensaries remained mindful of enfranchised White voters who might be angered by installing privies in schools, even White schools. They remained mindful of planters and mill owners in their districts who considered disease and deaths of workers part of doing business. While contemporary public health projects avoid practices of Jim Crow racism, they similarly conceal or fail to address current forms of structural racism and other social inequities that operate as key social determinants of health (Bonilla-Silva 1999; Garcia and Sherif, 2015; Williams and Mohammed, 2013). Public health communities continue to court political and elite donor support to pursue projects while side-stepping contentious issues of suppressed voting power, inequitable allocation of tax dollars, and unequal access to resources that might fulfill basic health needs. During the COVID-19 epidemic, they often hesitated to challenge business owners, politicians, MDs, and voters who eschewed a need for community-level containment—or any public health action—to the detriment of local communities of color (Bonilla-Silva 2022; Hotez 2021; Richardson et al., 2021; Tan et al., 2022).

Black public health reformers and Black-led community, university, and professional groups a century ago acted with a different vision and approach to public health, putting in place more comprehensive ranges of services. They were less-focused on single diseases and more on the sources of diseases, the totality of “lethal diseases” (Roman 1917, p. 66) and the social determinants of diseases, thereby centering the goals of “better farms, better homes, better education, and better health” (Beardsley 1987, pp.102-103). They used education and messaging, building grass-roots alliances to do so, while calling for equitable distributions of public resources. This vision of public health advanced more service-oriented, socially inclusive program activities, such as by the Tuskegee Woman’s Club and the Movable School, to remediate poor living conditions and environmental risks at the neighborhood level. Their activities over the early twentieth century institutionalized city-, state-, and nation-wide networks of rural and urban public health, nursing, and hospital services, under the “separate but equal” rules of the Jim Crow South. They leave a legacy of possibility, of sustained community-based efforts supportive of racial/ethnic-inclusion, even amidst organized sociopolitical and public health practices that historically legitimized their exclusion.

## Footnotes

1 The original 1900 Death Registration states were Connecticut, Indiana, Maine, Massachusetts, Michigan, New Hampshire, New Jersey, New York, Rhode Island, Vermont, plus the District of Columbia and some urban areas not in the Registration states. For historical background on U.S. Registration states and Vital Statistics collections see Linder and Grove (1943).

2 Trask (1916) compared Black mortality rates—measured in the U.S. Registration States—to mortality rates in other sampled countries finding them not to be enormously higher.

3 The southern plantation system in 1910 held nearly half of U.S. farms. Plantations were “continuous tract[s] of land…[under] control of a single individual or firm, all or a part of such tract being divided into at least five smaller tracts, which are leased to tenants” (U.S. Census Bureau 1916, p. 13). In most counties holding plantations, “Negroes constituted at least half of the total population” (U.S. Census Bureau 1916, p. 16).

4 For example, in Arkansas, the 1874 State Constitution weakened state Executive power by reducing a Governor’s term to two-years, allowing legislative over-rule of a Governor by majority-only vote, and constitutionally fixing the maximum state tax rate, thereby limiting revenue for expenditures (Goss 2011).

5 Tennessee Department of Public Health Records, 1879–1982, Record Group 97. Microfilm Processed by Gregory T. Yates, Archival Technical Services, Tennessee State Library and Archives July 26, 1995. http://tsla.tnsosfiles.com.s3.amazonaws.com/history/state/recordgroups/findingaids/DEPARTMENT_OF_PUBLIC_HEALTH_RECORDS_1879-1982.pdf Accessed December 23, 2023.

6 Born in Slavery: Slave Narratives from the Federal Writers’ Project, 1936 to 1938. Volume 2, Arkansas, Part 1. Lizzie Barnet, interviewed by Rosa Ingram in Conway, Arkansas. Library of Congress. https://wwvw.loc.gov/resource/mesn.021/?st=pdf&pdfPage=3 Accessed May 14, 2024.

7 Development of Rural Health Services. *100 Years: The Rockefeller Foundation*, excerpt from Trustees Bulletin December 1, 1939. Rockefeller Foundation records RG 3.1, Series 908, Box 14, Folder 149, Rockefeller Archive Center, Sleepy Hollow, NY [Hereafter RSC at RAC].

8 Helminth *Necator Americanus* (hookworm) requires high average temperatures and is rarely observed north of latitude 52° N. Transmission occurs when hatched larvae from eggs buried in sandy, contaminated soil penetrate the skin of a human host through direct contact with feet or hands. For overviews of parasitic infections see (historical) Faust (1955) and (contemporary) Hotez (2008).

9 Letter from Wickliffe Rose to F. T. Gates. October 24, 1911. Rockefeller Sanitary Commission Series 1, Reel 01, Box 1, Folder 28 (microform). RSC at RAC.

10 John D. Rockefeller funded the Rockefeller Foundation (RF) in June 1909 and RSC in October 1909. In June 1913, after the RF obtained New York State Senate approval for operations, the International Health Commission (IHC) extended eradication of hookworm efforts so that “… [the Rockefeller] Sanitary Commission would continue its work in the American South, while the Health Commission would divide its work among …Latin America, and in British colonies in the Far East and in the West Indies.” (Farley 2004, p. 4; Chapter 4 for hookworm activities 1913–1920). The IHC tracked hookworm in U.S. southern states after the RSC disbanded in 1914 but its primary focus was international (Browm 1976; Farley 2004).

11 Rose continued as head of the GEB, without pay, during his RSC appointment; four RSC Board appointees also served on the GEB. The GEB coordinated and monitored northern foundations funding Black education in the 1900s–1940s South (Anderson 1988; Anderson and Moss, 1999; Du Bois 2007 [1903]).

12 Letter from Wickliffe Rose to Dr. William Allen. March 22, 1911. Series 1, Box 1, Folder 11 (microform). RSC at RAC.

13 U.S. sovereignty is partitioned among federal, state, and local jurisdictions; weak southern states blocked federal power (as states-rights claims) and concentrated power at the local level, closer to powerful planters’ political control (James 1988). Local concentration of power with uneven contestation by non-planter White urban elites produced differential treatment of interest groups—such as social reformers—by local elected officials across counties, leading to uneven policy implementation across local jurisdictions (James 1988; Malczewski 2011; Werum 1999).

14 This long history of southern MD resentment was not assuaged by another 1910 philanthropic effort, involving a Carnegie-supported report by Alexander Flexner—brother of RSC Administrative Board member Simon Flexner—that declared American medical education not obtained through evidence-based university training—primarily offered in northern universities—to be inadequate if not quackery (Duffy 2011).

15 Letter from Dr. Arthur McCormack to Wickliffe Rose, enclosed report by Dr. Richmond. May 4, 1912. Series 2, Reel M 05, Box 6, Folder 114, Kentucky January-July 1912 (Microform). RSC at RAC.

16 Medical News. *Journal of the American Medical Association*, March 18, 1911: 824.

17 Letter from Wickliffe Rose to Dr. J. LaBruce Ward. July 12, 1911. Series 2, Reel M 08, Box 10, Folder 167 (microform). Also Letter from Dr. W. S. Leathers to Wickliffe Rose. June 10, 1911. Series 2, Reel M 06, Box 8, Folder 132 (microform). RSC at RAC.

18 Letter from Dr. Morgan Smith to Wickliffe Rose. June 9, 1911. Series 2, Reel M 03, Box 4, Folder 85 (Microform). RSC at RAC.

19 Letter from Dr. Morgan Smith to Wickliffe Rose. August 28, 1911. Series 2, Reel M 03, Box 4, Folder 85 (Microform). RSC at RAC.

20 Dispensary work in Mississippi. Dr. W. S. Leathers. Undated. Series 2, Reel M 06, Box 8, Folder 138 (Microform). RSC at RAC.

21 Letter, Dr. T. M. Fly. August 11, 1913. Series 2, Reel M 03, Box 4, Folder 87 (Microform). RSC at RAC.

22 Letter, Wickliffe Rose to Dr. W. S. Leathers. June 15, 1911. Series 2, Reel M 06, Box 7 F132 (Microform). RSC at RAC.

23 Letter September 10, 1913, from Dr. G. William Garrison to Dr. John Ferrell. Series 2, Reel M 03, Box 4, Folder 88 (Microform). RSC at RAC.

24 Letter September 13, 1913, from Dr. John Ferrell to Dr. William G. Garrison. Series 2, Reel M 03, Box 4, Folder 88 (Microform). RSC at RAC.

25 Letter April 10, 1911, from Olin West to Wickliffe Rose Folder 173 Tennessee (Microform). RSC at RAC.

26 Letter May 24, 1939, from B. E. Washburn to J. Ferrell Folder 33 (Microform). RSC at RAC.

27 Letter from Dr. Olin West to Dr. Wickliffe Rose. January 8, 1911. Folder 173 (Tennessee-General), Box 11, Reel 8 (Microform). RSC at RAC.

28 Letter from Wickliffe Rose to Dr. William Dinsmore. June 17, 1912. Series 2, Reel M 03, Box 4, Folder 79 (Microform). RSC at RAC.

29 Dr. John Kenney, medical director of the hospital at Tuskegee, quoted in Smith (1995, p. 28).

30 Judson, distinguishing full partnership and mediation, notes Black women’s groups used local racism to “further their own agenda, during a period and in a city where the possibilities for black political protest were severely limited” (1999, p. 94).

31 Judson describes the “interracial social etiquette” that required Black reformers—who were leaders in their communities—to communicate with White leadership using communication/behavioral signals consistent with an acceptance of racialized subordination (1999, p. 101; also see Schultz 2005).

32 Butler Center for Arkansas Studies, Arkansas State Bureau of Health Collection, Malaria and Malaria Control, Collection# MSS 97-33, Box 1 File 13, Special Bulletin #4, Malaria, undated.

33 Butler Center for Arkansas Studies, Arkansas State Bureau of Health Collection, Malaria and Malaria Control, Collection# MSS 97-33, Box 1 File 13, Special Bulletin #4, Malaria, undated.

34 Letter from Wickliffe Rose to Dr. William Dinsmore. June 17, 1912. Series 2, Reel M 03, Box 4, Folder 79 (Microform). RSC at RAC.

35 Global South plantation field workers were primarily non-White (Anderson 2002; Brown 1976; Farley 2004). In 1913, the IHC was finding considerable heterogeneity in hookworm prevalence by country, and by ethnicity within-country (Farley 2004). This interested the philanthropists and there was also archival chatter and debate about relative U.S. Black-White hookworm prevalence in letters, amidst a handful of differing back-of-the-envelope-style estimates and small localized reports. RSC administrators more often voiced the belief that hookworm prevalence was higher in the southern White than Black population. However, RSC Administrator Ferrell believed “the problem of hookworm disease is a problem of sanitation and as such does not admit of color line” (Jones et al., 1912, p. 311) while RSC Administrator Stiles (1915) documented higher Black than White rates in a community sample. The RSC had the scientific-medical resources in the U.S. southern field to examine race/ethnic differences in disease prevalence, as Black physicians requested, even for preliminary study. Thomas (2011, p. 13) suggests Rose avoided systematic collection of race-related statistics. Currently, U.S. hookworm prevalence is highest in predominantly Black U.S. communities with poor sanitation (Flowers 2022).

36 About half (49%) of counties in the eleven states had not funded dispensaries by August 1914. Planning for the project’s December termination, RSC administrators hastily compiled a list of non-participant counties to visit in 1915, even appropriating “$200 (for each)…to be used… where it is not feasible to secure the usual appropriation” (John A. Ferrell. Recommendation of Plan for Closing the Work of the Rockefeller Sanitary Commission in the Southern States. August 23, 1914. Series 1, Reel M 01, Box 2, Folder 33 (Microform). RSC at RAC.

37 Letter from Wickliffe Rose to Dr. William Dinsmore. June 17, 1912. Series 2, Reel M 03, Box 4, Folder 79 (Microform). RSC at RAC.
